# Estimation of loss of genetic diversity in modern Japanese cultivars by comparison of diverse genetic resources in Asian pear (*Pyrus* spp.)

**DOI:** 10.1186/s12863-016-0380-7

**Published:** 2016-06-14

**Authors:** Sogo Nishio, Norio Takada, Toshihiro Saito, Toshiya Yamamoto, Hiroyuki Iketani

**Affiliations:** Institute of Fruit Tree and Tea Science, NARO (NIFTS), 2-1 Fujimoto, Tsukuba, Ibaraki 305-8605 Japan; Department of Biosphere-Geosphere Science, Faculty of Biosphere-Geosphere Science, Okayama University of Science, 1-1 Ridai-cho, Kita-ku, Okayama 700-0005 Japan

**Keywords:** Simple sequence repeat (SSR) markers, *Pyrus pyrifolia* Nakai, Population structure, Principal coordinate analysis (PCoA)

## Abstract

**Background:**

Pears (*Pyrus* spp.) are one of the most important fruit crops in temperate regions. Japanese pear breeding has been carried out for over 100 years, working to release new cultivars that have good fruit quality and other desirable traits. Local cultivar ‘Nijisseiki’ and its relatives, which have excellent fruit texture, have been repeatedly used as parents in the breeding program. This strategy has led to inbreeding within recent cultivars and selections. To avoid inbreeding depression, we need to clarify the degree of inbreeding among crossbred cultivars and to introgress genetic resources that are genetically different from modern cultivars and selections. The objective of the present study was to clarify the genetic relatedness between modern Japanese pear cultivars and diverse Asian pear genetic resources.

**Results:**

We genotyped 207 diverse accessions by using 19 simple sequence repeat (SSR) markers. The heterozygosity and allelic richness of modern cultivars was obviously decreased compared with that of wild individuals, Chinese pear cultivars, and local cultivars. In analyses using Structure software, the 207 accessions were classified into four clusters (*K* = 4): one consisting primarily of wild individuals, one of Chinese pear cultivars, one of local cultivars from outside the Kanto region, and one containing both local cultivars from the Kanto region and crossbred cultivars. The results of principal coordinate analysis (PCoA) were similar to those from the Structure analysis. Wild individuals and Chinese pears appeared to be distinct from other groups, and crossbred cultivars became closer to ‘Nijisseiki’ as the year of release became more recent.

**Conclusions:**

Both Structure and PCoA results suggest that the modern Japanese pear cultivars are genetically close to local cultivars that originated in the Kanto region, and that the genotypes of the modern cultivars were markedly biased toward ‘Nijisseiki’. Introgression of germplasm from Chinese pear and wild individuals that are genetically different from modern cultivars seems to be key to broadening the genetic diversity of Japanese pear. The information obtained in this study will be useful for pear breeders and other fruit breeders who have observed inbreeding depression.

**Electronic supplementary material:**

The online version of this article (doi:10.1186/s12863-016-0380-7) contains supplementary material, which is available to authorized users.

## Background

Inbreeding is a common problem in fruit breeding programs [1–9]. It reduces vigor trait, such as tree vigor [2, 3, 5, 8], viability [1, 2, 5] and fruit weight [4]. Selection of local cultivars from wild populations during domestication has increased productivity but narrowed genetic diversity [10, 11]. Moreover, an organized breeding program is generally started with limited genetic resources that have been already domesticated, such as local cultivars. Introducing materials from foreign countries is not easy on account of biosecurity concerns, and introgression of superior traits from wild individuals may require several generations to reach performance comparable to that of modern cultivars. In addition, fruit trees have a long juvenile period before fruit set and seed production. Thus, most fruit breeders are likely to improve genotypes by crossing well-known cultivars [6, 12]. As a result, the genetic diversity of modern cultivars has decreased dramatically over time.

Pears (*Pyrus* spp.) belong to the subtribe Pyrinae (formerly subfamily Maloideae) of the Rosaceae and are one of the most important fruit crops in temperate regions. In East Asia, major cultivated pears are traditionally classified into three species: *P. ussuriensis* Maxim., *P. bretschneideri* Rehder, and *P. pyrifolia* (Burm. f.) Nakai [13, 14]. Many taxonomists and horticulturists have tried to classify these species using their own criteria, according to morphological characters [13, 15–17] or molecular markers [18–21]. However, these species readily produce interspecific hybrids [22], and some cultivars are admixtures of different species [18–21]. Also, the species themselves seem to be genetically continuous [15, 23–25]. As a result, genetic classification based on these three species is obscure. Iketani et al. [25] proposed a new cultivar classification system based on the population structure of these species and historical enumeration, i.e., *Pyrus* Ussurian Pear Group (*Pyrus ussuriensis*), *Pyrus* Chinese White Pear Group (*P. bretschneideri* or *P. pyrifolia*), *Pyrus* Chinese Sand Pear Group (*P. pyrifolia*), and *Pyrus* Japanese Pear Group (*P. pyrifolia*). Many pear genetic diversity studies have been conducted using these various systems to classify species, groups, and cultivars. However, few studies have focused on comparison of modern cultivars with diverse genetic resources such as foreign cultivars, wild individuals, and local cultivars.

The Japanese pear breeding program began in 1909 [26] and continues to aim at developing new cultivars that ripen at various times and have high productivity and fruit quality, low production costs, high disease resistance, self-compatibility, and freedom from physiological disorders. In particular, breeding for soft fruit texture has been key to improving fruit quality [27]. Local cultivar ‘Nijisseiki’, which originated in the Kanto region and has been one of the leading cultivars in Japan, has excellent fruit texture. ‘Nijisseiki’ and its relatives have been repeatedly used as parents in the breeding program. In addition, ‘Osanijisseiki’, which arose as a natural mutant from ‘Nijisseiki’, was released as a self-compatible cultivar and introduced into the breeding program as a means of developing additional self-compatible cultivars [28, 29]. For these reasons, inbreeding due to repeated use of ‘Nijisseiki’-biased genotypes has become a problem for Japanese pear breeding [8, 9]. It was reported that both the pedigree-based inbreeding coefficient (*F*) and the marker-based inbreeding coefficient increased in Japanese pear cultivars as the year of the initial cross became more recent [9]. The tree height of 1-year-old seedlings decreased by 20 % for *F* = 0.25 and by 40 % for *F* = 0.5 [8]. Also, the decrease in number of *S*-genotypes among modern cultivars is problematic with respect to mating design. These days, more and more cultivars and selections have identical *S*-genotypes, with the result that some combinations among modern cultivars are incompatible. A possible solution may lie in the diverse genetic resources (including cultivars introduced from China, local cultivars collected from all over Japan, and wild individuals) that have been preserved at Institute of Fruit Tree and Tea Science, NARO (NIFTS). We are interested in using these genetic resources to avoid inbreeding depression.

So far, the use of foreign cultivars and wild individuals in Japanese pear breeding has been limited. It is extremely difficult to obtain elite genotypes in a short period of time while at the same time broadening genetic diversity using foreign cultivars and wild individuals instead of well-adapted genotypes. To overcome this difficulty, it is necessary to genetically characterize these materials so as to determine which cultivars or individuals would be most effective for broadening genetic diversity and how much genetic diversity has been lost among modern cultivars. The objective of the present study was to clarify the degree of inbreeding among modern cultivars and to estimate the genetic relatedness between modern cultivars and diverse genetic resources. On the basis of our results, we discuss the potential to broaden genetic diversity in pear breeding programs and the trend toward loss of genetic diversity in modern pear cultivars.

## Methods

### Plant materials

The nine groups (207 accessions) used in this study are shown in Table 1 and Additional file 1: Table S1. Several of the materials in this study are similar to those used in Iketani et al. [25]: wild individuals of *P. ussuriensis* collected from the Hayasaka-Kogen high plateau in Iwate Prefecture (IWA), which were unaffected by the genetic influence of cultivated trees [25]; Chinese pear cultivars generally considered to be *P. bretschneideri* (BRE) and *P. ussuriensis* (USS); and Japanese pear local cultivars that originated in the Kanto region of Japan (KAN), near the Sea of Japan (NSJ), and in western Japan (WJ). We also included Japanese pear crossbred cultivars and breeding lines from the first half of the 20th century (CFH), the latter half of the 20th century (CLH), and the 21st century (MDC).

**Table 1:** List of the 207 pear accessions used in this study

ID	Cultivar/selection	Type	Code	ID	Cultivar/selection	Type	Code
			(group number)				(group number)
1	Hs-1	Wild	IWA (1)	106	Babaucchiaginashi	Local cultivar	WJ (6)
2	Hs-2	Wild	IWA (1)	107	Ichihara Wase	Local cultivar	WJ (6)
3	Hs-3	Wild	IWA (1)	108	Imamuraaki	Local cultivar	WJ (6)
4	Hs-4	Wild	IWA (1)	109	Imamuranatsu	Local cultivar	WJ (6)
5	Hs-5	Wild	IWA (1)	110	Nansei Chabo	Local cultivar	WJ (6)
6	Hs-6	Wild	IWA (1)	111	Nekogoroshi	Local cultivar	WJ (6)
7	Hs-7	Wild	IWA (1)	112	Sawairiyamanashi	Local cultivar	WJ (6)
8	Hs-8	Wild	IWA (1)	113	Segawa	Local cultivar	WJ (6)
9	Hs-9	Wild	IWA (1)	114	Shimokatsuginashi	Local cultivar	WJ (6)
10	Hs-10	Wild	IWA (1)	115	Shoumyoujinashi	Local cultivar	WJ (6)
11	Hs-11	Wild	IWA (1)	116	Tosajou	Local cultivar	WJ (6)
12	Hs-12	Wild	IWA (1)	117	Tosajounishiki	Local cultivar	WJ (6)
13	Hs-14	Wild	IWA (1)	118	Tosanashi	Local cultivar	WJ (6)
14	Hs-15	Wild	IWA (1)	119	Tsukushiinunashi	Local cultivar	WJ (6)
15	Hs-16	Wild	IWA (1)	120	Waseaka Ouryuu	Local cultivar	WJ (6)
16	Hs-18	Wild	IWA (1)	121	Atago	Crossbred cultivar	CFH (7)
17	Hs-19	Wild	IWA (1)	122	Ishii Wase	Crossbred cultivar	CFH (7)
18	Baozhuli	Cultivar	BRE (2)	123	Higashino	Crossbred cultivar	CFH (7)
19	Chang Xi Li	Cultivar	BRE (2)	124	Heiwa	Crossbred cultivar	CFH (7)
20	Hong Li	Cultivar	BRE (2)	125	Gion	Crossbred cultivar	CFH (7)
21	Hong Xiao Li	Cultivar	BRE (2)	126	Kikusui	Crossbred cultivar	CFH (7)
22	Huang Li	Cultivar	BRE (2)	127	Sagami	Crossbred cultivar	CFH (7)
23	Mi Li	Cultivar	BRE (2)	128	Seiryuu	Crossbred cultivar	CFH (7)
24	Mi Li Cui	Cultivar	BRE (2)	129	Yakumo	Crossbred cultivar	CFH (7)
25	Tai Huang Li	Cultivar	BRE (2)	130	Niitaka	Crossbred cultivar	CFH (7)
26	Ya Gua Li	Cultivar	BRE (2)	131	Asahi	Crossbred cultivar	CFH (7)
27	Ya Li	Cultivar	BRE (2)	132	Yachiyo	Crossbred cultivar	CFH (7)
28	Kuerren Xiang Li	Cultivar	BRE (2)	133	Hatsuaki	Crossbred cultivar	CFH (7)
29	Lunanhuangli	Cultivar	BRE (2)	134	Kimizukawase	Crossbred cultivar	CFH (7)
30	Ma Ke Zao Li	Cultivar	BRE (2)	135	Kougetsu	Crossbred cultivar	CFH (7)
31	Man Yuan Xiang	Cultivar	BRE (2)	136	Hattatsu	Crossbred cultivar	CFH (7)
32	Ping Li	Cultivar	BRE (2)	137	Shinkou	Crossbred cultivar	CFH (7)
33	Seuri Li	Cultivar	BRE (2)	138	Shinseiki	Crossbred cultivar	CFH (7)
34	Xie Hua Tian	Cultivar	BRE (2)	139	Seigyoku	Crossbred cultivar	CFH (7)
35	Yin Bai Li	Cultivar	BRE (2)	140	Yanaga	Crossbred cultivar	CFH (7)
36	Yuan Ba Li	Cultivar	BRE (2)	141	Shinsetsu	Crossbred cultivar	CLH (8)
37	Wo Wo Li	Cultivar	BRE (2)	142	Hiratsuka 7	Breeding line	CLH (8)
38	Suan Li	Cultivar	BRE (2)	143	Hiratsuka 1	Breeding line	CLH (8)
39	Tang Li	Cultivar	BRE (2)	144	Hiratsuka 11	Breeding line	CLH (8)
40	Dang Shan Fu Su Li	Cultivar	BRE (2)	145	Kumoi	Crossbred cultivar	CLH (8)
41	Ba Li Xiang	Cultivar	USS (3)	146	Suisei	Crossbred cultivar	CLH (8)
42	Bei Jin Bai Li	Cultivar	USS (3)	147	Kousui	Crossbred cultivar	CLH (8)
43	Cang Xi Li	Cultivar	USS (3)	148	Hiratsuka 10	Breeding line	CLH (8)
44	Dang Shan Jin Gai Su	Cultivar	USS (3)	149	Hiratsuka 17	Breeding line	CLH (8)
45	Dang Shan Mian Li	Cultivar	USS (3)	150	Hiratsuka 24	Breeding line	CLH (8)
46	Dang Shan Zi Su Li	Cultivar	USS (3)	151	Shinsui	Crossbred cultivar	CLH (8)
47	Huang Shan Li	Cultivar	USS (3)	152	Hayatama	Crossbred cultivar	CLH (8)
48	Hui Zhou Xue Li	Cultivar	USS (3)	153	Tama	Crossbred cultivar	CLH (8)
49	Jian Ba Li	Cultivar	USS (3)	154	Housui	Crossbred cultivar	CLH (8)
50	Lai Yang Ci Li	Cultivar	USS (3)	155	Hiratsuka 25	Breeding line	CLH (8)
51	Manshuu Yaseinashi	Cultivar	USS (3)	156	Hiratsuka 29	Breeding line	CLH (8)
52	Niao Li	Cultivar	USS (3)	157	Hiratsuka 27	Breeding line	CLH (8)
53	Ping Guo Li	Cultivar	USS (3)	158	Hakkou	Crossbred cultivar	CLH (8)
54	Su Hyang Ri	Cultivar	USS (3)	159	Chouju	Crossbred cultivar	CLH (8)
55	Zao Su	Cultivar	USS (3)	160	Hokukan	Crossbred cultivar	CLH (8)
56	Zhu Zui Li	Cultivar	USS (3)	161	Tsukuba 34	Breeding line	CLH (8)
57	Doitsu	Local cultivar	KAN (4)	162	Tsukuba 35	Breeding line	CLH (8)
58	Choujuurou	Local cultivar	KAN (4)	163	Tsukuba 37	Breeding line	CLH (8)
59	Nijisseiki	Local cultivar	KAN (4)	164	Tsukuba 39	Breeding line	CLH (8)
60	Yoshino	Local cultivar	KAN (4)	165	Shinsei	Crossbred cultivar	CLH (8)
61	Edoya	Local cultivar	KAN (4)	166	Shuugyoku	Crossbred cultivar	CLH (8)
62	Rokugatsu	Local cultivar	KAN (4)	167	Chikusui	Crossbred cultivar	CLH (8)
63	Okuroku	Local cultivar	KAN (4)	168	Yasato	Crossbred cultivar	CLH (8)
64	Jouhana	Local cultivar	KAN (4)	169	Nansui	Crossbred cultivar	CLH (8)
65	Heishi	Local cultivar	KAN (4)	170	Tsukuba 41	Breeding line	CLH (8)
66	Wase Kouzou	Local cultivar	KAN (4)	171	Tsukuba 42	Breeding line	CLH (8)
67	Kouzou	Local cultivar	KAN (4)	172	Tsukuba 43	Breeding line	CLH (8)
68	Shikishima	Local cultivar	KAN (4)	173	Tsukuba 44	Breeding line	CLH (8)
69	Shinchuu	Local cultivar	KAN (4)	174	Wakahikari	Crossbred cultivar	CLH (8)
70	Rikiya	Local cultivar	KAN (4)	175	Hougetsu	Crossbred cultivar	CLH (8)
71	Chousen	Local cultivar	KAN (4)	176	Natsuhikari	Crossbred cultivar	CLH (8)
72	Shirayuki	Local cultivar	KAN (4)	177	Nikkori	Crossbred cultivar	CLH (8)
73	Kokuchou	Local cultivar	KAN (4)	178	Akibae	Crossbred cultivar	CLH (8)
74	Taihei	Local cultivar	KAN (4)	179	Akemizu	Crossbred cultivar	CLH (8)
75	Sekiryuu	Local cultivar	KAN (4)	180	Nangetsu	Crossbred cultivar	CLH (8)
76	Taihaku	Local cultivar	KAN (4)	181	Hokushin	Crossbred cultivar	CLH (8)
77	Sekaiichi	Local cultivar	KAN (4)	182	Inagi	Crossbred cultivar	CLH (8)
78	Asahiryuu	Local cultivar	KAN (4)	183	Aikansui	Crossbred cultivar	CLH (8)
79	Kinchaku	Local cultivar	KAN (4)	184	Kisui	Crossbred cultivar	CLH (8)
80	Koyuki	Local cultivar	KAN (4)	185	Yoshikaori	Crossbred cultivar	CLH (8)
81	Saitama 2-1	Local cultivar	KAN (4)	186	Tsukuba 52	Breeding line	MDC (9)
82	Saitama 8	Local cultivar	KAN (4)	187	Tsukuba 53	Breeding line	MDC (9)
83	Amanogawa	Local cultivar	NSJ (5)	188	Tsukuba 49	Breeding line	MDC (9)
84	Ruisannashi	Local cultivar	NSJ (5)	189	Tsukuba 51	Breeding line	MDC (9)
85	Okusankichi	Local cultivar	NSJ (5)	190	Akizuki	Crossbred cultivar	MDC (9)
86	Hakuteiryuu	Local cultivar	NSJ (5)	191	Nashi Chukanbohon Nou 1 Gou	Crossbred cultivar	MDC (9)
87	Abumi	Local cultivar	NSJ (5)	192	Akiakari	Crossbred cultivar	MDC (9)
88	Yokogoshi	Local cultivar	NSJ (5)	193	Oushuu	Crossbred cultivar	MDC (9)
89	Awayuki	Local cultivar	NSJ (5)	194	Shuurei	Crossbred cultivar	MDC (9)
90	Hachibuse No Nashi	Local cultivar	NSJ (5)	195	Natsushizuku	Crossbred cultivar	MDC (9)
91	Oohiromaru	Local cultivar	NSJ (5)	196	Shinkansen	Crossbred cultivar	MDC (9)
92	Kounowatashi	Local cultivar	NSJ (5)	197	Kanta	Crossbred cultivar	MDC (9)
93	Miyadani	Local cultivar	NSJ (5)	198	Rinka	Crossbred cultivar	MDC (9)
94	Onba	Local cultivar	NSJ (5)	199	Hatsumaru	Crossbred cultivar	MDC (9)
95	Shimane Yamanashi	Local cultivar	NSJ (5)	200	Hoshiakari	Crossbred cultivar	MDC (9)
96	Hakataao	Local cultivar	NSJ (5)	201	Tsukuba 59	Breeding line	MDC (9)
97	Kunitomi	Local cultivar	NSJ (5)	202	Tsukuba 60	Breeding line	MDC (9)
98	Nishitonami 1	Local cultivar	NSJ (5)	203	Tsukuba 61	Breeding line	MDC (9)
99	Ookoga	Local cultivar	NSJ (5)	204	Tsukuba 62	Breeding line	MDC (9)
100	Shihyakume	Local cultivar	NSJ (5)	205	Tsukuba 63	Breeding line	MDC (9)
101	Tanponashi	Local cultivar	NSJ (5)	206	Tsukuba 64	Breeding line	MDC (9)
102	Touhou	Local cultivar	NSJ (5)	207	Narumi	Crossbred cultivar	MDC (9)
103	Tottori 4	Local cultivar	NSJ (5)				
104	Waseaka	Local cultivar	NSJ (5)				
105	Yagoemon	Local cultivar	NSJ (5)				

*IWA* Wild individuals from Iwate, *BRE P. bretschneideri* cultivar, *USS P. ussuriensis* cultivar, *KAN* Local cultivar from Kanto region, *NSJ* Local cultivar from near the sea of Japan, *WJ* Local cultivar from western Japan, *CFH* Cultivar released in the first half of the 20th century, *CLH* Cultivar released in the latter half of the 20th century or breeding line developed during that time, *MDC* Modern cultivar (released in the 21st century) or breeding line developed during that time

### SSR marker analysis

The 207 pear accessions were genotyped for 19 simple sequence repeat (SSR) markers (Additional file 2: Table S2). PCR amplification was performed in 10 μL containing 5 μL of 2× Green GoTaq reaction buffer (0.4 mM each dNTP, 3 mM MgCl_2_, and 1 U *Taq* polymerase, pH 8.5, Promega, Madison, USA), 20 pmol of each forward primer labeled with a fluorescent chemical (FAM or HEX) and unlabeled reverse primer, and 2.5 ng of genomic DNA. Amplification was performed in 35 cycles of 94 °C for 1 min, 55 °C for 1 min, and 72 °C for 2 min. PCR products were separated and detected with a 3130 xl genetic analyzer (Life Technologies Co., Carlsbad, CA, USA). The size of each amplified band was determined by comparison with an internal DNA standard (400HD-ROX, Life Technologies) in GeneScan software (Life Technologies).

### Data analyses

The observed heterozygosity (*H*_O_) and expected heterozygosity (*H*_E_) were calculated in GenAlEx v. 6.5 software [30], and allelic richness (AR, *n* =15) was calculated in FSTAT v. 2.9.3 software [31]. Bayesian statistical inference on the population structure was performed in Structure v. 2.3.3 software [32] with the admixture model for ancestry and both independent and correlated models for allele frequency, without any prior information about the origin of each individual. After a burn-in period of 100,000 iterations, the analysis was run 10 times for each value of *K* (number of inferred ancestral populations) from 2 to 10 for 1,000,000 iterations. We used Evanno et al.’s [33] criterion of |L″(*K*)| = |L′(*K* + 1) – L′(*K*)| = |lnP (X|*K* + 1) – 2lnP(X|*K* + 1) + lnP(X|*K* + 1)| and ∆*K* = mean (|L″(*K*)|)/s[L(*K*)] and values of plateaued lnP(X|*K*) to estimate the optimal value of *K*. Simulation studies have shown that once the real value of *K* has been reached, lnP(X|K) will typically plateau or continue to increase slightly [33]. Principal coordinate analysis (PCoA) was calculated in GenAlEx 6.5 from the pairwise genetic distances obtained with the covariance-standardized method. Simple allele-sharing distances among the 207 accessions were calculated as described [34]. All data were calculated from the genotypes of the 207 accessions based on the 19 SSR markers.

## Results

### Basic genetic characteristics of pear accession groups

We genotyped nine groups, consisting of 207 accessions, by using 19 SSR markers. Heterozygosity of the nine groups was *H*_O_ = 0.42–0.74 and *H*_E_ = 0.39–0.80 (Table 2). Crossbred cultivar groups released after 1950 (CLH and MDC) had lower values than the other 7 groups. AR of the nine groups ranged from 3.2 to 9.6. AR of the Chinese pear groups (BRE and USS) showed the highest values (8.0 and 9.6) among the nine groups. On the other hand, AR of the crossbred cultivar groups (CFH, CLH, and MDC) decreased as the year of release became more recent (4.6, 3.5, and 3.2, respectively). The AR values of MDC were about half those of the local cultivar groups (KAN, NSJ, WJ).

**Table 2:** Genetic characteristics of group analyzed using 19 SSRs

Group number	Code	*H*_O_	*H*_E_	AR
1	IWA	0.69	0.71	6.4
2	BRE	0.72	0.75	8.0
3	USS	0.74	0.80	9.6
4	KAN	0.72	0.66	5.1
5	NSJ	0.68	0.71	6.1
6	WJ	0.66	0.71	6.3
7	CFH	0.69	0.61	4.6
8	CLH	0.54	0.47	3.5
9	MDC	0.42	0.39	3.2

*H*_O_ observed heterozygosity, *H*_E_ expected heterozygosity, AR allelic richness

### Bayesian statistical inference of the population structure

**Fig. 1:**
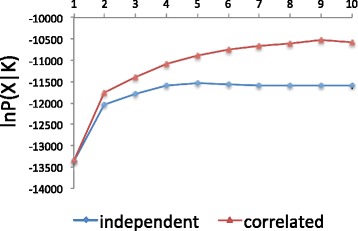
Values of lnP(X|K) for values of *K* from 1 to 10

**Fig. 2:**
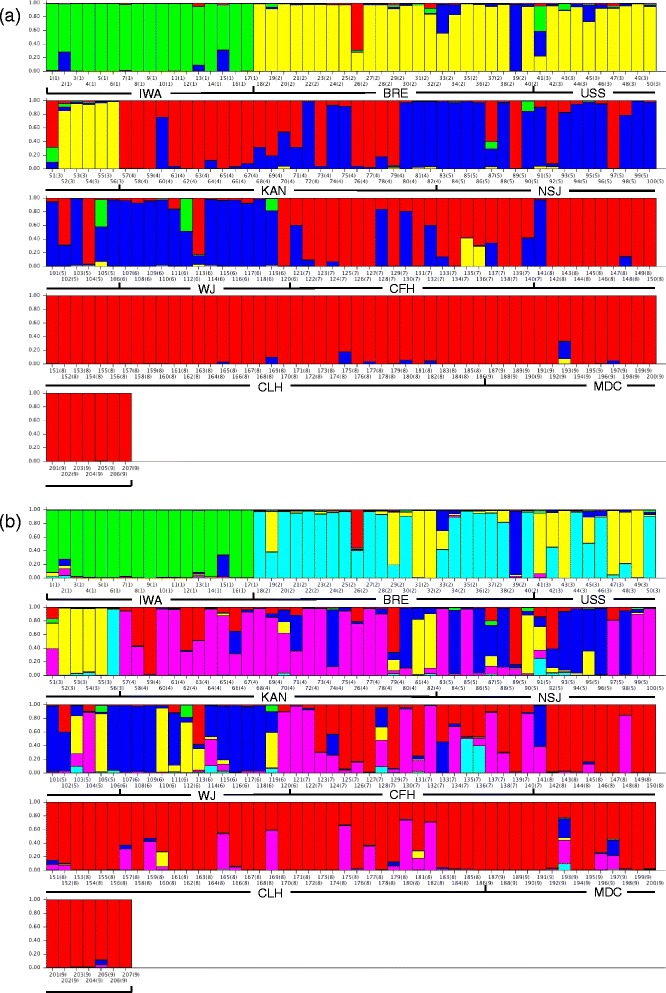
A detailed bar plot diagram for (**a**) *K* = 4 in the independent model and (**b**) *K* = 6 in the correlated model. The first number under each bar represents the individual accession ID number (1–207); the second number (in parentheses) represents the group number (1–9). ID numbers and groups are defined in Table 1

To estimate the optimal number of genetic clusters (*K*) in Structure, we calculated ∆*K* values (Table 3). The ∆*K* values were highest at *K* = 2 in both the independent and correlated models. In both models, the two clusters corresponded to (1) wild individuals and Chinese pear groups (IWA, BRE, and USS) and (2) local and crossbred cultivar groups (KAN, NSJ, WJ, CFH, CLH and MDC), similar to the classification obtained by Iketani et al. [24]. In the independent model, the second-highest ∆*K* value occurred at *K* = 4 (Table 3). Moreover, the value of lnP(X|K) seemed to plateau at *K* = 4 (Fig. 1). Consequently we adopted *K* = 4 as the optimal classification in the independent model. With *K* = 4, the 207 accessions could be classified into four groups corresponding to (1) wild individuals from Iwate Prefecture (green cluster), (2) Chinese pear cultivars (yellow cluster), (3) local cultivars from the Kanto region and crossbred cultivars (red cluster), and (4) local cultivars from outside the Kanto region (blue cluster; Fig. 2a). We were not able to separate the local cultivars from the Kanto region from the crossbred cultivars by increasing the value of *K* in the independent model. The new clusters that appeared at *K* = 6 to 10 were distributed mainly in the Chinese pear cultivars.

**Table 3:** Values of Δ*K* for *K* = 1 to 10 in independent and correlated models

*K*	Δ*K*	
	Independent	Correlated
1	–	–
2	1646.7	1610.6
3	14.3	6.9
4	102.3	2.4
5	53.5	4.1
6	2.4	1.5
7	4.1	2.0
8	1.6	1.9
9	2.6	2.1
10	–	–

On the other hand, no prominent ∆*K* was observed in the correlated model other than for *K* = 2 (Table 3), and the value of lnP(X|*K*) seemed to plateau somewhere between *K* = 6 and *K* = 10, although it did not plateau as clearly as for the independent model (Fig. 1). However, we confirmed that the bar plot diagrams at *K* = 6 (Fig. 2b) were similar across ten repetitions, suggesting that the clustering at *K* = 6 in the correlated model has high reliability. The difference in genetic structure between *K* = 4 in the independent model and *K* = 6 in the correlated model was the appearance of a new cluster in the Chinese pear groups (cyan) and in the local cultivar group from the Kanto region (magenta; Fig. 2). At *K* = 6, some accessions showed admixtures of different clusters (suggesting contributions from different populations), which was observed much less frequency in the *K* = 4 classification. In particular, some crossbred cultivars had characteristics of both the red and magenta clusters when *K* = 6, which we attribute to cross-hybridization between genotypes from different clusters in the pear breeding program. Among the local cultivars from the Kanto region (KAN), only ‘Nijisseiki’ (ID = 59) was dominated by the red cluster at *K* = 6, probably because cultivars released in the 20th century and later are based on ‘Nijisseiki’ and its relatives.

### PCoA of the 207 accessions

**Fig. 3:**
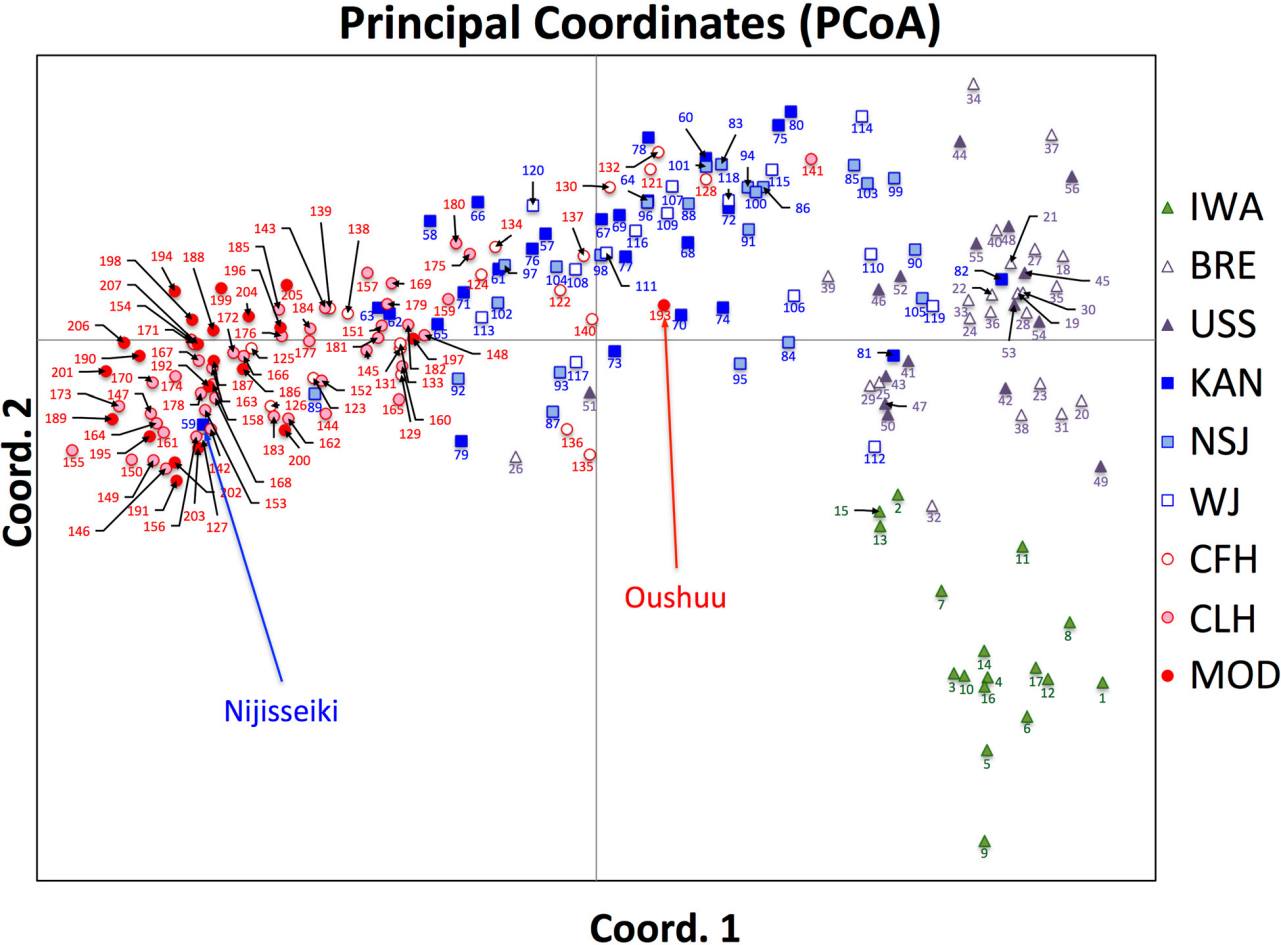
Principal coordinate analysis (PCoA) plot generated from genetic distance calculations among the 207 accessions in GenAlEx software. Group codes are as shown in Table 1

The first two informative PCo components (Fig. 3) explained 23.15 % of the total variation. The results of PCoA were similar to those of the Structure analysis. The wild individual group (IWA) and Chinese pear groups (BRE, USS) appeared to be distinct from the other groups. BRE and USS showed similar distribution, as did NSJ and WJ. Crossbred cultivars (CFH, CLH, MDC) were plotted closer to ‘Nijisseiki’ (ID = 59) as the year of release became more recent. In particular, all of the MDC cultivars except ‘Oushuu’ (ID = 193) were distributed near ‘Nijisseiki’. Some cultivars showed unexpected distributions; for example, Chinese pear cultivars ‘Ya Gua Li’ (ID = 26) and ‘Manshuu Yaseinashi’ (ID = 51) were plotted between the Japanese local cultivars and the Chinese pear cultivars. ‘Saitama 2-1’ (ID = 81) and ‘Saitama 8’ (ID = 82), both of which belong to the group of local cultivars from the Kanto region, were close to the Chinese pear groups.

We also calculated simple allele-sharing distances to estimate the genetic relationship between each pair of accessions (Additional file 3: Table S3). The average simple allele-sharing distances were 0.22, 0.30, 0.54 and 0.73 between MDC and wild *P. ussuriensis* (IWA), Chinese pear cultivars (BRE and USS), local cultivars (KAN, NSJ, and WJ), and crossbred cultivars (CFH, CLH, and MDC), respectively.

## Discussion

### Genetic relationships between modern Japanese pear cultivars and diverse genetic resources

We evaluated diverse genetic resources of Asian pear using Structure analysis, PCoA, and simple allele-sharing distance. The results of Structure analysis and PCoA were consistent in that wild individuals and Chinese pear cultivars were classified as genetically distinct from Japanese pear cultivars. This result is also in good agreement with previous studies [24, 25]. The modern cultivars seemed to be genetically close to local cultivars that originated in the Kanto region, especially ‘Nijisseiki’ (Figs. 2 and 3). Because modern cultivars were selected from ‘Nijisseiki’ and its relatives, this result was not unexpected. Both low heterozygosity and low AR were observed in Japanese pear modern cultivars, suggesting that the genetic diversity of modern cultivars has decreased over time. In the Structure result with *K* = 4, almost all crossbred cultivars and modern cultivars showed “red” genetic background (i.e., were part of the cluster indicated by red shading in Fig. 2), which was characteristic of local cultivars from the Kanto region. When *K* = 6, the “red” genetic background was shared by only ‘Nijisseiki’ and modern cultivars. In addition, almost all of the modern cultivars were plotted around ‘Nijisseiki’ in PCoA, and the average allele-sharing distance between modern cultivars and ‘Nijisseiki’ was high (0.76; Additional file 3: Table S3). These results suggest a marked biased in the genotypes of modern cultivars. Crossing among modern cultivars containing only the “red” genetic background would not generate a genotype that greatly exceeds the current performance of modern cultivars. To broaden genetic diversity and obtain superior genotypes in modern cultivars, we need to introduce genes from wild individuals and Chinese pear cultivars that are genetically different from modern Japanese pear cultivars.

### Breeding history of ‘Oushuu’, a cultivar with a diverse genetic background

Among the modern cultivars, only ‘Oushuu’ (ID = 193) seemed to be distinct from the others in both Structure analysis and PCoA. This cultivar, released in 2003, is an offspring of a cross between C2 (an offspring of a cross between ‘Lai Yang Ci Li’ (ID = 50) and ‘Nijisseiki’) and ‘Shinsetsu’ (ID = 141, an offspring of a cross between ‘Imamuraaki’ (ID = 108) and ‘Okusankichi’ (ID = 85)). ‘Oushuu’ showed both strong tree vigor and desirable fruit texture characteristics (e.g., soft flesh firmness) preferred in the Japanese market [35]. In the Structure result with *K* = 6, ‘Oushuu’ had not only “red” genetic background (presumably from ‘Nijisseiki’) but also “cyan” (presumably from ‘Lai Yang Ci Li’), “blue” (presumably from ‘Imamuraaki’), and “magenta” (presumably from ‘Okusankichi’) genetic backgrounds. Its strong tree vigor may be caused by heterosis. The successful breeding history of ‘Oushuu’ indicates that it is possible to release new cultivars that have good fruit quality without repeatedly using ‘Nijisseiki’ and its relatives as parents.

### Cultivars showing unexpected genetic structure

Some cultivars showed unexpected genetic structure and genetic relationships. ‘Ya Gua Li’ (ID = 26) and ‘Manshuu Yaseinashi’ (ID = 51) were classified into the Chinese pear group [25], but were plotted between Japanese local cultivars and Chinese pear cultivars in PCoA. ‘Ya Gua Li’ appears to have the genetic structure of both Chinese pear and local cultivars from the Kanto region. In fact, ‘Ya Gua Li’ and ‘Nijisseiki’ shared at least one allele at each of the 19 SSR markers, suggesting that ‘Ya Gua Li’ is a hybrid between Chinese pear and ‘Nijisseiki’. This hybrid could have been mislabeled during genetic resource preservation. ‘Manshuu Yaseinashi’ had admixed genetic structure, but the results with *K* = 4 and *K* = 6 were different, i.e., it contained “blue” genetic background (found in Japanese local cultivars) when *K* = 4 and “yellow” genetic background (found in Chinese pear cultivars) when *K* = 6. Because the word “Manshuu” is the old name of a place in northeastern China and “Yaseinashi” means “wild pear” in Japanese, wild individuals from northeastern China will be key to clarifying the true genetic structure of ‘Manshuu Yaseinashi’. Although ‘Tang Li’ (ID =39) was classified as BRE, it has an almost totally “blue” genetic background (characteristic of Japanese local cultivars) at both *K* = 4 and *K* = 6. Pear genetic resources are generally grafted onto seedling rootstocks of Japanese materials such as strain ‘Mamenashi’, the collective name of wild pear with small fruit. Thus, it is possible that ‘Tang Li’ had been mislabeled or mishandled during genetic resource preservation and is actually a Japanese rootstock genotype. We need to take care because these cultivars showing unexpected genetic structure are not appropriate for broadening the genetic diversity of Japanese pear cultivars. ‘Saitama 2-1’ (ID = 81) and ‘Saitama 8’ (ID = 82) are considered to be local cultivars from the Kanto region, because “Saitama” is the name of a prefecture in the Kanto region, but these cultivars were genetically close to the Chinese cultivar group in PCoA. As with ‘Manshuu Yaseinashi’, the Structure results for ‘Saitama 2-1’ and ‘Saitama 8’ were inconsistent between *K* = 4 and *K* = 6. Further analyses including verified Chinese materials are needed to identify the origin of these cultivars.

### Putative spreading patterns of local cultivars

Local cultivars that originated outside the Kanto region seem to have been important contributors to the genetic diversity of cultivated pears. It has been reported that cultivars that originated near the Sea of Japan or Kyushu island tend to show late ripening and long fruit, whereas cultivars that originated in the Kanto region show early ripening and oblate fruit [36, 37]. However, some local cultivars that originated outside the Kanto region (‘Awayuki’ (ID = 89), ‘Kounowatashi’ (ID = 92), ‘Kunitomi’ (ID = 97), ‘Touhou’ (ID = 102), and ‘Waseaka’ (ID = 104)) showed genetic background similar to those that originated in the Kanto region at *K* = 4, suggesting that these cultivars might have been introduced from the Kanto region into other regions. In fact, it was reported that ‘Waseaka’ was introduced into Niigata Prefecture from the Kanto region, and ‘Kunitomi’ was discovered from offspring of ‘Taihaku’ (ID = 76), which originated in the Kanto region [26]. Similarly, these other cultivars might have been introduced from the Kanto region into other regions. In fruit tree species, it is common that local cultivars with traits of interest are vegetatively propagated and carried to other regions [38–41]. We need to take into account the spread of cultivars when classifying local cultivars by geographical origin.

### Possible origin of local cultivars in Japan

Iketani et al. [24] showed that local cultivars in Japan are genetically closer to Chinese cultivars than to wild individuals of *P. ussuriensis* collected from high plateaus in Iwate Prefecture. In the Structure result at *K* = 6, some local cultivars in Japan showed “yellow” genetic background, characteristic of Chinese pear cultivars. The history of pear breeding before the early modern period in Japan is still unclear, but some local cultivars may have been domesticated from Chinese materials. Jiang et al. [42] suggested that there were opportunities for ancient cultivar exchange between Japan and eastern China. Thus, the “yellow” genetic background might have been introduced by genetic exchange with Chinese cultivars.

### Future breeding strategy for the Japanese pear breeding program

Our data strongly support the assumption that introgression of germplasm from Chinese pear cultivars and wild individuals into modern cultivars is an effective way to broaden genetic diversity. Thus, it might be easier to develop new cultivars using Chinese pear cultivars, which are already domesticated and bear large fruit. In fact, ‘Oushuu’ was selected from the first backcross of a Chinese cultivar to several Japanese cultivars and has good fruit quality. On the other hand, introgression of germplasm from indigenous species into modern cultivars seems to be challenging; for example, their fruit size is smaller than that of recent cultivars [43]. However, there are many hybrids between *P. pyrifolia* and *P. ussuriensis*, some of which bear fruits larger than those of wild individuals of the latter species [44]. In particular, local cultivar ‘Natsunashi’ would be good material for a pear breeding program because it shows early ripening and has high concentrations of ethyl and methyl esters, which are desirable flavor components.

### Trends in genetic diversity during organized breeding of annual and perennial crops

Owing to the progress of organized breeding (scientific breeding), the genetic diversities of several annual crops, including maize [45, 46], rice [47], sorghum [48], tobacco [49], and wheat [50], decreased at one point during the latter part of the 20th century. According to a meta-analysis of genetic diversity trends in annual crops during the 20th century, diversity was reduced significantly in the 1960s compared with the 1950s, than then recovered from the 1970s to the 1990s [51]. Breeders probably averted the narrowing of the germplasm base and subsequently increased the genetic diversity in these crops through the introgression of novel materials. However, very few genetic studies have focused on genetic diversity trends during the organized breeding of perennial crops, although inbreeding depression has been a concern of fruit breeders [6, 51]. Our results clarify that loss of genetic diversity has occurred in a fruit crop, as has been reported in annual crops. However, the genetic diversity in perennial crops seems to have declined more slowly than that in annual crops, possibly because perennial crops have a longer juvenile phase and fewer sexual cycles than annual crops. Japanese pear breeding may have just reached the stage reached by annual crop breeding in the 1960s or 1970s. Given the availability of genetic resources and molecular tools, we now have the capability to work toward increasing the genetic diversity of pear cultivars.

## Conclusions

In this study, we clarified the genetic relationship between Japanese pear modern cultivars and diverse genetic resources including wild individuals, Chinese pear cultivars, and local cultivars. The genetic diversity of modern cultivars decreased as the year of release became more recent. The modern cultivars were genetically close to local cultivar ‘Nijisseiki’, which had been repeatedly used as a parent in our breeding program, confirming that Japanese pear breeding has been carried out within a narrow gene pool. On the basis of these findings, we plan to broaden the genetic diversity in the NIFTS pear breeding program by introgressing germplasm from Chinese pear and wild individuals that are genetically distinct from modern cultivars. We also determined that Structure analysis and PCoA are effective for evaluating the degree of inbreeding and genetic relationships among accessions. The information obtained in this study will be useful for pear breeders as well as other fruit breeders who have problems with inbreeding depression in their breeding programs.

## Abbreviations

AR, allelic richness; BRE, Chinese pear cultivars generally considered to be *P. bretschneideri*; CFH, Japanese pear crossbred cultivars and breeding lines from the first half of the 20th century; CLH, Japanese pear crossbred cultivars and breeding lines from the latter half of the 20th century; *H*_E_, expected heterozygosity; *H*_O_, observed heterozygosity; IWA, wild individuals of *P. ussuriensis* collected from the Hayasaka-Kogen high plateau in Iwate Prefecture; KAN, Japanese pear local cultivars that originated in the Kanto region of Japan; MDC, Modern cultivar released in the 21st century or breeding line developed during that time; NIFTS, Institute of Fruit Tree and Tea Science, NARO; NSJ, Japanese pear local cultivars that originated near the Sea of Japan; PCoA, principal coordinate analysis; SSR, simple sequence repeat; USS, Chinese pear cultivars generally considered to be *P. ussuriensis*; WJ, Japanese pear local cultivars that originated in western Japan

## Additional files

Additional file 1: Table S1. Names, accession numbers, and breeding information for the 207 accessions used in this study. (PDF 154 kb) Additional file 2: Table S2. Description of the 19 simple sequence repeat (SSR) markers used in this study. (XLSX 12 kb) Additional file 3: Table S3. Simple allele-sharing distances for the 207 accessions used in this study. (XLSX 259 kb)
