# Supplementary material for: Estimation of loss of genetic diversity in modern Japanese cultivars by comparison of diverse genetic resources in Asian pear (Pyrus spp.)
Source: BMC Genet. 2016 Jun 14;17:81. doi: 10.1186/s12863-016-0380-7 (PMC4908778; doi:10.1186/s12863-016-0380-7)
Supplement: Additional file 1: Table S1. — Names, accession numbers, and breeding information for the 207 accessions used in this study. (PDF 154 kb) [file 12863_2016_380_MOESM1_ESM.pdf]

Table S1 Names, accession numbers, and breeding information for the 207 accessions used in this study.

ID	Cultivar/selection	Type	Code (group number)	Accession no.	Generation	Year of release	Original name
1	Hs-1	Wild	IWA (1)	JP244361			
2	Hs-2	Wild	IWA (1)				
3	Hs-3	Wild	IWA (1)				
4	Hs-4	Wild	IWA (1)				
5	Hs-5	Wild	IWA (1)				
6	Hs-6	Wild	IWA (1)				
7	Hs-7	Wild	IWA (1)				
8	Hs-8	Wild	IWA (1)				
9	Hs-9	Wild	IWA (1)				
10	Hs-10	Wild	IWA (1)				
11	Hs-11	Wild	IWA (1)				
12	Hs-12	Wild	IWA (1)				
13	Hs-14	Wild	IWA (1)				
14	Hs-15	Wild	IWA (1)				
15	Hs-16	Wild	IWA (1)				
16	Hs-18	Wild	IWA (1)				
17	Hs-19	Wild	IWA (1)				
18	Baozhuli	Cultivar	BRE (2)	JP118544			宝珠梨
19	Chang Xi Li	Cultivar	BRE (2)	JP113751			昌渓梨
20	Hong Li	Cultivar	BRE (2)	JP113733			紅梨
21	Hong Xiao Li	Cultivar	BRE (2)	JP113734			紅宵梨
22	Huang Li	Cultivar	BRE (2)	JP113750			黄梨
23	Mi Li	Cultivar	BRE (2)	JP113753			蜜梨
24	Mi Li Cui	Cultivar	BRE (2)	JP113759			蜜梨脆
25	Tai Huang Li	Cultivar	BRE (2)	JP113760			胎黄梨
26	Ya Gua Li	Cultivar	BRE (2)	JP113740			鴉爪梨
27	Ya Li	Cultivar	BRE (2)	JP113741			鴨梨
28	Kuerren Xiang Li	Cultivar	BRE (2)	JP116292			庫爾勒香梨
29	Lunanhuangli	Cultivar	BRE (2)	JP118545			魯南黄梨
30	Ma Ke Zao Li	Cultivar	BRE (2)	JP113744			麻売早梨
31	Man Yuan Xiang	Cultivar	BRE (2)	JP116293			満園香
32	Ping Li	Cultivar	BRE (2)	JP113754			平梨
33	Seuri Li	Cultivar	BRE (2)	PI541904			
34	Xie Hua Tian	Cultivar	BRE (2)	JP113755			謝花甜
35	Yin Bai Li	Cultivar	BRE (2)	JP113757			銀白梨
36	Yuan Ba Li	Cultivar	BRE (2)	JP113742			円把梨
37	Wo Wo Li	Cultivar	BRE (2)	JP113739			凹凹梨
38	Suan Li	Cultivar	BRE (2)	JP116295			酸梨
39	Tang Li	Cultivar	BRE (2)	JP113820			棠梨
40	Dang Shan Fu Su Li	Cultivar	BRE (2)				碭山伏酥梨
41	Ba Li Xiang	Cultivar	USS (3)	JP113749			八里香
42	Bei Jin Bai Li	Cultivar	USS (3)	JP113731			北京白梨
43	Cang Xi Li	Cultivar	USS (3)	JP113752			蒼渓梨
44	Dang Shan Jin Gai Su	Cultivar	USS (3)	JP116288			碭山金盖酥
45	Dang Shan Mian Li	Cultivar	USS (3)	JP116289			碭山面梨
46	Dang Shan Zi Su Li	Cultivar	USS (3)	JP116290			碭山紫酥梨
47	Huang Shan Li	Cultivar	USS (3)	JP116291			黄山梨
48	Hui Zhou Xue Li	Cultivar	USS (3)	JP115734			灰州雪梨
49	Jian Ba Li	Cultivar	USS (3)	JP113735			尖把梨
50	Lai Yang Ci Li	Cultivar	USS (3)	JP113736			莱陽慈梨
51	Manshuu Yaseinashi	Cultivar	USS (3)	JP113821			満州野生梨
52	Niao Li	Cultivar	USS (3)	JP115739			鳥梨
53	Ping Guo Li	Cultivar	USS (3)	JP116294			苹果梨
54	Su Hyang Ri	Cultivar	USS (3)	JP113738			水香梨
55	Zao Su	Cultivar	USS (3)	JP116298			早酥
56	Zhu Zui Li	Cultivar	USS (3)	JP113743			猪嘴梨
57	Doitsu	Local cultivar	KAN (4)	JP113577			独逸
58	Choujuurou	Local cultivar	KAN (4)	JP113574			長十郎
59	Nijisseiki	Local cultivar	KAN (4)	JP113631			二十世紀
60	Yoshino	Local cultivar	KAN (4)	JP113692			吉野
61	Edoya	Local cultivar	KAN (4)	JP113578			江戸屋
62	Rokugatsu	Local cultivar	KAN (4)	JP113642			六月
63	Okuroku	Local cultivar	KAN (4)	JP113633			晩六
64	Jouhana	Local cultivar	KAN (4)	JP113606			上花
65	Heishi	Local cultivar	KAN (4)	JP113592			平子
66	Wase Kouzou	Local cultivar	KAN (4)	JP113682			早生幸蔵
67	Kouzou	Local cultivar	KAN (4)	JP113620			幸蔵
68	Shikishima	Local cultivar	KAN (4)	JP113654			敷島
69	Shinchuu	Local cultivar	KAN (4)	JP113656			真鍮
70	Rikiya	Local cultivar	KAN (4)	JP113641			力弥
71	Chousen	Local cultivar	KAN (4)	JP113576			朝鮮
72	Shirayuki	Local cultivar	KAN (4)	JP113663			白雪
73	Kokuchou	Local cultivar	KAN (4)	JP113621			国長
74	Taihei	Local cultivar	KAN (4)	JP113667			大平
75	Sekiryuu	Local cultivar	KAN (4)	JP113651			赤龍
76	Taihaku	Local cultivar	KAN (4)	JP113666			太白
77	Sekaiichi	Local cultivar	KAN (4)	JP113650			世界一
78	Asahiryuu	Local cultivar	KAN (4)	JP113569			旭竜
79	Kinchaku	Local cultivar	KAN (4)	JP113613			巾着
80	Koyuki	Local cultivar	KAN (4)	JP113622			小雪
81	Saitama 2-1	Local cultivar	KAN (4)	JP113806			埼玉2-1
82	Saitama 8	Local cultivar	KAN (4)	JP113809			埼玉8
83	Amanogawa	Local cultivar	NSJ (5)	JP113562			天の川
84	Ruisannashi	Local cultivar	NSJ (5)	JP113643			類三梨
85	Okusankichi	Local cultivar	NSJ (5)	JP113634			晩三吉
86	Hakuteiryuu	Local cultivar	NSJ (5)	JP113586			伯帝龍
87	Abumi	Local cultivar	NSJ (5)	JP113559			鐙
88	Yokogoshi	Local cultivar	NSJ (5)	JP113691			横越
89	Awayuki	Local cultivar	NSJ (5)	JP113572			淡雪
90	Hachibuse No Nashi	Local cultivar	NSJ (5)	JP113786			鉢伏の梨
91	Oohiromaru	Local cultivar	NSJ (5)	JP113637			大広丸
92	Kounowatashi	Local cultivar	NSJ (5)	JP113616			耕渡
93	Miyadani	Local cultivar	NSJ (5)	JP239686			宮谷
94	Onba	Local cultivar	NSJ (5)	JP113636			オンバ
95	Shimane Yamanashi	Local cultivar	NSJ (5)	JP113810			島根山梨
96	Hakataao	Local cultivar	NSJ (5)	JP113584			博多青
97	Kunitomi	Local cultivar	NSJ (5)	JP113624			国富
98	Nishitonami 1	Local cultivar	NSJ (5)	JP113785			西砺波-1
99	Ookoga	Local cultivar	NSJ (5)	JP113638			大古河
100	Shihyakume	Local cultivar	NSJ (5)	JP113653			四百匁
101	Tanponashi	Local cultivar	NSJ (5)	JP113669			タンポナシ
102	Touhou	Local cultivar	NSJ (5)	JP113671			登報
103	Tottori 4	Local cultivar	NSJ (5)	JP113815			鳥取在来-4
104	Waseaka	Local cultivar	NSJ (5)	JP113678			早生赤
105	Yagoemon	Local cultivar	NSJ (5)	JP113687			弥五右衛門
106	Babaucchiaginashi	Local cultivar	WJ (6)	JP113763			ババウッチャギナシ
107	Ichihara Wase	Local cultivar	WJ (6)	JP113599			市原早生
108	Imamuraaki	Local cultivar	WJ (6)	JP113600			今村秋
109	Imamuranatsu	Local cultivar	WJ (6)	JP113601			今村夏
110	Nansei Chabo	Local cultivar	WJ (6)	JP115741			南勢チャボ
111	Nekogoroshi	Local cultivar	WJ (6)	JP113628			猫殺
112	Sawairiyamanashi	Local cultivar	WJ (6)	JP118551			サワイリヤマナシ
113	Segawa	Local cultivar	WJ (6)	JP113646			瀬川
114	Shimokatsuginashi	Local cultivar	WJ (6)	JP113662			霜被梨
115	Shoumyoujinashi	Local cultivar	WJ (6)	JP113685			称名寺梨
116	Tosajou	Local cultivar	WJ (6)	JP113672			土佐上
117	Tosajounishiki	Local cultivar	WJ (6)	JP113673			土佐条錦
118	Tosanashi	Local cultivar	WJ (6)	JP113674			土佐梨
119	Tsukushiinunashi	Local cultivar	WJ (6)	JP113777			ツクシイヌナシ
120	Waseaka Ouryuu	Local cultivar	WJ (6)	JP113680			早生赤黄龍
121	Atago	Crossbred cultivar	CFH (7)	JP113570	1st generation	1915	愛宕
122	Ishii Wase	Crossbred cultivar	CFH (7)	JP113603	1st generation	1921	石井早生
123	Higashino	Crossbred cultivar	CFH (7)	JP113594	1st generation	1922	東野
124	Heiwa	Crossbred cultivar	CFH (7)	JP113593	1st generation	1926	平和
125	Gion	Crossbred cultivar	CFH (7)	JP113582	1st generation	1927	祇園
126	Kikusui	Crossbred cultivar	CFH (7)	JP113611	1st generation	1927	菊水
127	Sagami	Crossbred cultivar	CFH (7)	JP113644	1st generation	1927	相模
128	Seiryuu	Crossbred cultivar	CFH (7)	JP113649	1st generation	1927	青竜
129	Yakumo	Crossbred cultivar	CFH (7)	JP113688	1st generation	1927	八雲
130	Niitaka	Crossbred cultivar	CFH (7)	JP113630	1st generation	1927	新高
131	Asahi	Crossbred cultivar	CFH (7)	JP113568	2nd generation	1931	旭
132	Yachiyo	Crossbred cultivar	CFH (7)	JP113686	1st generation	1935	八千代
133	Hatsuaki	Crossbred cultivar	CFH (7)	JP113588	1st generation	1939	初秋
134	Kimizukawase	Crossbred cultivar	CFH (7)	JP113612	1st generation	1940	君塚早生
135	Kougetsu	Crossbred cultivar	CFH (7)	JP113615	1st generation	1940	光月
136	Hattatsu	Crossbred cultivar	CFH (7)	JP113590	1st generation	1940	八達
137	Shinkou	Crossbred cultivar	CFH (7)	JP113657	1st generation	1941	新興
138	Shinseiki	Crossbred cultivar	CFH (7)	JP113658	1st generation	1945	新世紀
139	Seigyoku	Crossbred cultivar	CFH (7)	JP113647	1st generation	1931	清玉
140	Yanaga	Crossbred cultivar	CFH (7)	JP113689	1st generation	1935	弥長
141	Shinsetsu	Crossbred cultivar	CLH (8)	JP113659	1st generation	1949	新雪
142	Hiratsuka 7	Breeding line	CLH (8)		2nd generation	1949	リ-14
143	Hiratsuka 1	Breeding line	CLH (8)		2nd generation	1949	イ-33
144	Hiratsuka 11	Breeding line	CLH (8)		2nd generation	1949	オ-9
145	Kumoi	Crossbred cultivar	CLH (8)	JP113623	2nd generation	1955	雲井
146	Suisei	Crossbred cultivar	CLH (8)	JP113665	2nd generation	1955	翠星
147	Kousui	Crossbred cultivar	CLH (8)	JP113619	2nd generation	1959	幸水
148	Hiratsuka 10	Breeding line	CLH (8)		3rd generation	1963	平塚16号
149	Hiratsuka 17	Breeding line	CLH (8)		3rd generation	1963	42-6
150	Hiratsuka 24	Breeding line	CLH (8)		3rd generation	1963	92-7
151	Shinsui	Crossbred cultivar	CLH (8)	JP113660	2nd generation	1965	新水
152	Hayatama	Crossbred cultivar	CLH (8)	JP113591	2nd generation	1968	早玉
153	Tama	Crossbred cultivar	CLH (8)	JP113668	3rd generation	1971	多摩
154	Housui	Crossbred cultivar	CLH (8)	JP113598	3rd generation	1972	豊水
155	Hiratsuka 25	Breeding line	CLH (8)		3rd generation	1972	101-106
156	Hiratsuka 29	Breeding line	CLH (8)		3rd generation	1972	75-23
157	Hiratsuka 27	Breeding line	CLH (8)		3rd generation	1972	48-96
158	Hakkou	Crossbred cultivar	CLH (8)	JP113585	3rd generation	1972	八幸
159	Chouju	Crossbred cultivar	CLH (8)	JP113575	3rd generation	1973	長寿
160	Hokukan	Crossbred cultivar	CLH (8)	JP143889	2nd generation	1978	北甘
161	Tsukuba 34	Breeding line	CLH (8)		4th generation	1982	筑波34号
162	Tsukuba 35	Breeding line	CLH (8)		3rd generation	1982	筑波35号
163	Tsukuba 37	Breeding line	CLH (8)		4th generation	1982	筑波37号
164	Tsukuba 39	Breeding line	CLH (8)		4th generation	1982	筑波39号
165	Shinsei	Crossbred cultivar	CLH (8)	JP113694	3rd generation	1984	新星
166	Shuugyoku	Crossbred cultivar	CLH (8)	JP113707	3rd generation	1988	秀玉
167	Chikusui	Crossbred cultivar	CLH (8)	JP113716	4th generation	1989	筑水
168	Yasato	Crossbred cultivar	CLH (8)	JP113718	4th generation	1990	八里
169	Nansui	Crossbred cultivar	CLH (8)		3rd generation	1990	南水
170	Tsukuba 41	Breeding line	CLH (8)		5th generation	1992	筑波41号
171	Tsukuba 42	Breeding line	CLH (8)		5th generation	1992	筑波42号
172	Tsukuba 43	Breeding line	CLH (8)		5th generation	1992	筑波43号
173	Tsukuba 44	Breeding line	CLH (8)		5th generation	1992	筑波44号
174	Wakahikari	Crossbred cultivar	CLH (8)	JP238266	4th generation	1992	若光
175	Hougetsu	Crossbred cultivar	CLH (8)	JP113720	4th generation	1994	豊月
176	Natsuhikari	Crossbred cultivar	CLH (8)	JP238262	4th generation	1995	なつひかり
177	Nikkori	Crossbred cultivar	CLH (8)	JP118540	4th generation	1996	にっこり
178	Akibae	Crossbred cultivar	CLH (8)	JP238254	3rd generation	1997	秋栄
179	Akemizu	Crossbred cultivar	CLH (8)	JP238253	4th generation	1997	あけみず
180	Nangetsu	Crossbred cultivar	CLH (8)	JP238261	3rd generation	1997	南月
181	Hokushin	Crossbred cultivar	CLH (8)	JP238257	3rd generation	1997	北新
182	Inagi	Crossbred cultivar	CLH (8)	JP113602	2nd generation	1962	稲城
183	Aikansui	Crossbred cultivar	CLH (8)		4th generation	1990	愛甘水
184	Kisui	Crossbred cultivar	CLH (8)	JP238266	4th generation	1990	喜水
185	Yoshikaori	Crossbred cultivar	CLH (8)		1st generation	1992	吉香
186	Tsukuba 52	Breeding line	MDC (9)		5th generation	1999	筑波52号
187	Tsukuba 53	Breeding line	MDC (9)		4th generation	1999	筑波53号
188	Tsukuba 49	Breeding line	MDC (9)		5th generation	1999	筑波49号
189	Tsukuba 51	Breeding line	MDC (9)		4th generation	1999	筑波51号
190	Akizuki	Crossbred cultivar	MDC (9)	JP118538	5th generation	2001	あきづき
191	Nashi Chukanbohon Nou 1 Gou	Crossbred cultivar	MDC (9)	JP238479	1st generation	2002	266-27
192	Akiakari	Crossbred cultivar	MDC (9)	JP118536	5th generation	2003	あきあかり
193	Oushuu	Crossbred cultivar	MDC (9)	JP118539	2nd generation	2003	王秋
194	Shuurei	Crossbred cultivar	MDC (9)	JP118537	5th generation	2003	秋麗
195	Natsushizuku	Crossbred cultivar	MDC (9)	JP230439	5th generation	2008	なつしずく
196	Shinkansen	Crossbred cultivar	MDC (9)		5th generation	2008	新甘泉
197	Kanta	Crossbred cultivar	MDC (9)		6th generation	2013	甘太
198	Rinka	Crossbred cultivar	MDC (9)		6th generation	2013	凛夏
199	Hatsumaru	Crossbred cultivar	MDC (9)		5th generation	2014	はつまる
200	Hoshiakari	Crossbred cultivar	MDC (9)		6th generation	2014	ほしあかり
201	Tsukuba 59	Breeding line	MDC (9)		6th generation	2015	筑波59号
202	Tsukuba 60	Breeding line	MDC (9)		7th generation	2015	筑波60号
203	Tsukuba 61	Breeding line	MDC (9)		7th generation	2015	筑波61号
204	Tsukuba 62	Breeding line	MDC (9)		7th generation	2015	筑波62号
205	Tsukuba 63	Breeding line	MDC (9)		6th generation	2015	筑波63号
206	Tsukuba 64	Breeding line	MDC (9)		5th generation	2015	筑波64号
207	Narumi	Crossbred cultivar	MDC (9)		5th generation	2015	なるみ

Accession no. is based on either the JP accession number of the NIAS GENEBANK, Japan, or the PI number of the National Plant Germplasm System, USDA.

Original name is designated by Japanese style typefaces.

IWA = Wild individuals from Iwate, BRE = P. bretschneideri cultivar, USS = P. ussuriensis cultivar, KAN = Local cultivar from Kanto region, NSJ = Local cultivar from near the sea of Japan, WJ = Local cultivar from western Japan, CFH = Cultivar released in the first half of the 20th century, CLH = Cultivar released in the latter half of the 20th century or breeding line developed during that time, MDC = Modern cultivar (released in the 21st century) or breeding line developed during that time
